# Application of GIS Technology-Supported Cross Media Fusion Method Based on Deep Learning in Landscape Performance Evaluation

**DOI:** 10.1155/2022/8339895

**Published:** 2022-09-08

**Authors:** Xiaoqing Liu, Juanfen Wang, Xiao Rui, Jizhi Zhang

**Affiliations:** Architecture and Art Design College, Southeast University Chengxian College, Nanjing, Jiangsu 210088, China

## Abstract

GIS technology can provide reasonable and sustainable data support for landscape planning and ecological development and make wetland landscape planning consider the spatial layout of landscape and the optimal allocation of resources more. The key technologies of cross media intelligence mainly focus on intelligent information retrieval, analysis and reasoning, knowledge map construction, and intelligent storage. Convolutional neural network (CNN), as one of the representative algorithms of deep learning, plays an important role in retrieving landscape data and extracting image and text features across media. Further retrieval of media data, in-depth text processing, and image feature data extraction are realized by using deep learning technology, and comprehensive in-depth analysis is carried out by combining landscape plane images, three-dimensional images, and vector information in GIS technology. Provide quantitative information for the evaluation system of human landscape, economy, history, and region, so as to formulate a scientific and reasonable performance evaluation system.

## 1. Introduction

In the process of practice, landscape performance evaluation often makes a comparison of the comprehensive evaluation of the space before and after the built environment. It includes the quantitative changes in traffic, safety, greening rate, and so on before and after the project practice, as well as people's evaluation on the use of space. There are also tracking descriptions of spatial performance changes within a certain time range. LAF is the earliest and most comprehensive landscape performance data research institution [1]. The platform mainly includes case briefing, performance tools, knowledge express, and academic achievements. The performance tools on LAF website have more than 30 benefit categories and put forward the calculation method of more than 100 indicators [1].

At present, the key technologies of cross media intelligence [2] mainly focus on intelligent information retrieval, analysis and reasoning, knowledge map construction, intelligent storage. such as cross media intelligent publications, archives information intelligent management system, smart city construction plan, internet intelligent information analysis, and so on. With the continuous integration of artificial intelligence technology in many fields, cross media intelligence has gradually been deeply applied in cross media intelligent processing, cross media intelligent data retrieval and sharing, educational activity platform design, and other fields.

**Figure 1:**
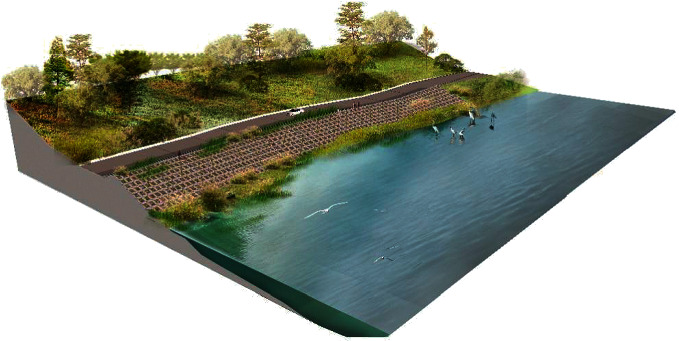
3D landscape map.

With the development of landscape ecology theory, the introduction of geographic information system (GIS) technology into landscape planning has become an important analysis tool for landscape planning research, which can provide reasonable and sustainable data support for landscape planning and ecological development and make landscape planning consider the spatial layout of landscape and the optimal allocation of resources more, as shown in Figure 1. Therefore, the application of GIS technology is more and more widely and deeply used in the development, utilization, and research of landscape architecture.

## 2. Literature Review

### 2.1. Landscape Performance

The authoritative literature on landscape performance research mainly focuses on the research level related to ecology, environment, and resources. In 2009, LAF proposed the term “landscape performance” during the creation of the landscape performance series (LPS) strategic research initiative. Landscape performance is based on measurable results to evaluate the environmental, social, and economic benefits of landscape. The term “landscape performance” is defined as “a measure of the efficiency of a landscape solution in meeting its preset goals while meeting sustainability.” In the definition, sustainability is one of the main objectives of design and planning.

**Figure 2:**
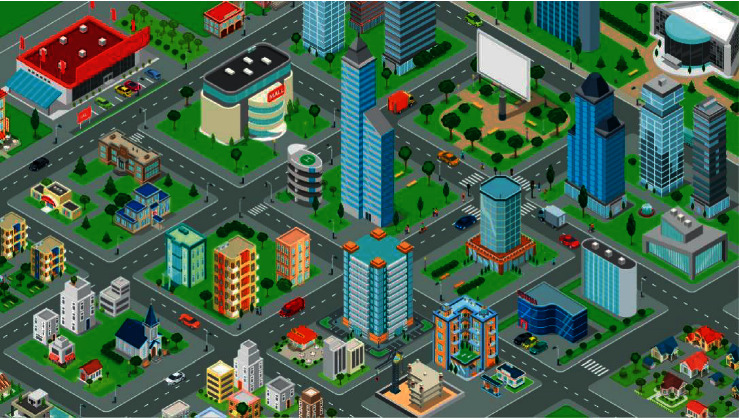
Urban 3D landscape.

The evaluation index system of landscape performance is based on the three elements of sustainable development, namely, good environment, social equity, and economic feasibility [3]. At the level of ecological benefit indicators, China's landscape performance evaluation lacks the calculation of groundwater, as shown in Figure 2. However, for the habitat part, China's assessment indicators have added habitat creation, protection, and restoration to adapt to China's specific case analysis. The purpose of measuring the social benefit indicators is to evaluate whether the implementation of the project contributes to the construction of social equity and improves the quality of space use at all levels. The landscape performance evaluation includes all evaluation indicators of LAF, and at the same time, it adds relevant indicators of jobs, tourist consumption, and economic growth.

At present, the main research methods used in landscape performance include use evaluation, case investigation and analysis, analytic hierarchy process, fuzzy comprehensive method, comprehensive index method, and so on [4].

### 2.2. Cross Media Intelligence

As the core technology in the era of artificial intelligence 2.0, cross media intelligence has unique advantages in intelligent retrieval, analytical reasoning, and knowledge construction. It provides technical support for multidimensional resource search, semantic analysis, and autonomous learning of educational games in multiple fields. Cross media intelligence should move from processing multimedia data by types to a new level of cross media cognition, learning, and reasoning.

In 2010, Davidson defined cross media as [5, 6] tightly mixing text, image, voice, video, and other media resources and their interactive attributes. Cross media is mainly reflected in the communication and interaction of information between different media. From the perspective of basic attributes, cross media is a new media form with three basic attributes: cross modal and cross platform attributes, rich expression and presentation attributes, and social attributes of media data. It obtains different forms of media information from a variety of channels0 and closely combines with relevant natural and social attribute information to vividly reflect the social behavior information of individuals and groups.

Scholars have summarized the concept from different angles. Combined with the era background of intelligence, it is proposed that cross media intelligence is an important part of the new generation of artificial intelligence [6]. Through the theories and methods of audio-visual perception, machine learning, and language computing, a unified semantic expression of the entity world is constructed. Through cross media analysis and reasoning, the data are transformed into intelligence, to make all kinds of information systems intelligent [7]. From the perspective of media form, the following definitions are given. The meaning of cross media intelligence is to complete the functions of recognition, reasoning, design, creation and prediction of related things by comprehensively utilizing the memory information of vision, language, hearing and other sensory organs [8, 9].

### 2.3. GIS

GIS, first proposed by Roger Tomlinson, is a system for analyzing and processing geographic data [10]. GIS can not only reflect the characteristics of the surface and underground but also analyze the two-dimensional and three-dimensional characteristics of the atmosphere, which solves the problem of analyzing and utilizing space resources. Analysis, evaluation, management, etc. are widely used. GIS has become a comprehensive system integrating computer science, geography, economics, administrative management, and other disciplines [11, 12].

In the early stage of GIS development, due to the limitation of technology, GIS can only provide modules that meet certain functions, and the modules lack coordination, so a complete system cannot be formed [13]. Until the development of computer technology and other technologies in the past three decades, GIS technology has gradually matured. Italy's CGIS and the US NASA National Space Laboratory's ELAS information system are relatively well-known GIS systems. Satellite navigation system is also a major thrust for the development of GIS technology.

The main support of GIS technology is computer and its related equipment. With the speed of computer processor getting faster and faster, the storage capacity is getting larger and larger. On the one hand, it has greatly promoted the development of GIS technology. On the other hand, it also makes the previous traditional GIS technology lag with the information and digital era. In recent years, GIS has shown the development trend of networking, openness, virtual reality, spatial multidimensional, etc. [14]. GIS also has many development directions. For example, Web GIS, which has developed rapidly in recent years, has strong adaptability, wide application fields, and simple operation. Users do not have to worry about the management and maintenance of the database, so that GIS is truly integrated into life, making it omnipotent and omnipresent [15]. In the computer and communication environment, Open GIS [16] is a GIS system established according to industry standards and interfaces, which is characterized by allowing data not only to be limited within the system but also to be transmitted between systems. Its interoperability and portability are unmatched by traditional GIS. Virtual GIS [17] is also a new direction of GIS development proposed with the development of VR technology in recent years. It combines GIS technology with VR technology to conduct advanced human-computer interaction under visual and auditory simulation. It is believed that VR technology is integrated into GIS technology, and VGIS will bring users unprecedented experience.

### 2.4. Deep Learning

Deep learning is a relatively young field in academia, which was first proposed in 2006. Deep learning belongs to the category of artificial intelligence research [18]. Deep learning, machine learning, and pattern recognition are three very similar concepts.

Deep learning is a branch of machine learning. At present, China has invested more resources in the field of external in-depth learning, and many breakthroughs in the application field have once again proved the price of in-depth learning. It can be said that deep learning has become the most intelligent learning method close to the human brain at present, which has caused competition among major manufacturers around the world. For example, Baidu and Alibaba in China and Google in foreign countries have all set foot in the field of deep learning [19].

Similar to human, machine learning is a process from unknown to known. If a machine has ability to solve a problem, with the increasing number of problems it solves, the performance of the machine or its ability to solve problems will be enhanced under the influence of the program. It can be said that this machine has learning ability [20]. The depth model needs a large amount of training data for training, and people inadvertently become trainers in life. The arrival of the big data era provides more resources for machine learning. For example, in the common speech recognition technology [21], the machine converts the received speech information into corresponding characters for display. Users can use this function at the same time. The model established by Reddy has become the basis of model training data. Many users and the proposed modifications have also become a rare free resource for enterprises.

## 3. Theories

### 3.1. Cross Media Retrieval Technology Based on Deep Learning

**Figure 3:**
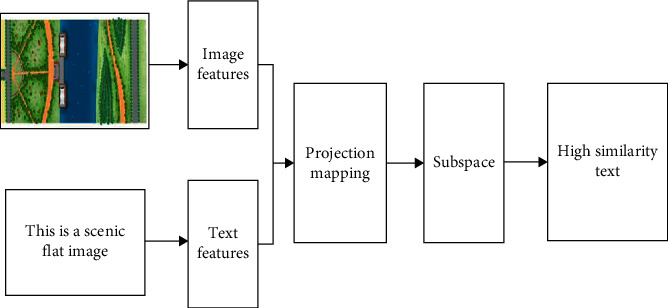
Cross media retrieval process.

In the face of huge and complex multimedia data, the traditional single media physical examination can no longer meet the needs of people's daily operation. Cross media retrieval technology came into being [22]. Through cross media retrieval technology [23], users only need to submit a media type data, and then they can obtain a variety of media data related to its content semantics. However, the heterogeneous gap between different media data brings great challenges to the realization of cross media retrieval. The implementation of cross media retrieval technology needs the support of a variety of technologies, including computer vision, natural language processing technology, and artificial intelligence, as shown in Figure 3.

In the above process, data feature extraction and projection mapping are particularly critical [23]. Revolutionary neural network (CNN) [24] is a deep feedforward artificial neural network and one of the representative algorithms of deep learning. CNN is also widely used in image, natural language processing, target detection, and other fields.

**Figure 4:**
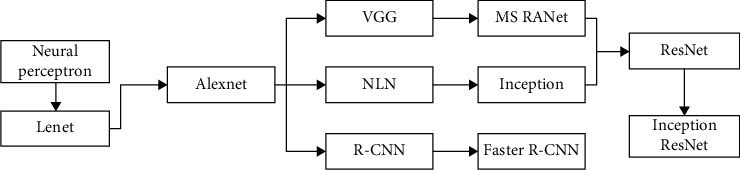
Development process of CNN.

The neural perceptron, proposed by Kunihiko Fukushima, is considered as the original prototype of CNN [25]. On this basis, subsequent researchers continued to innovate and introduced new algorithms to optimize the network, which greatly improved the expression ability of CNN. With the deepening of the network and the application of new algorithms, the performance of CNN has completely surpassed human beings in some aspects. CNN has also been expanded in more and more tasks. The development path of CNN is shown in Figure 4.

### 3.2. Integration of GIS Technology and Deep Learning

The basic task of image recognition is to identify one or more targets contained in the image through analysis [26]. Generally, image recognition mainly includes three links. The first step is image preprocessing, the second step is image feature extraction, and the third step is feature pattern classification. Among them, feature extraction determines the accuracy of the whole image recognition and is also the basis for distinguishing different image recognition methods.

**Figure 5:**
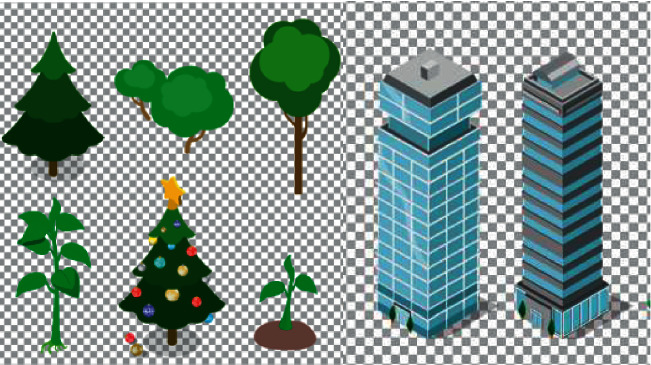
Landscape and building icons.

GIS icon symbols belong to two-dimensional images, and the icon symbols themselves have no noise and other interference information [27], as shown in Figure 5. In the image preprocessing stage, the icon symbol itself does not need to undergo conventional operations such as noise reduction. Most image recognition methods have no learning ability. The neural network method, which is the closest to the deep learning method, uses the neural network algorithm to recognize and classify images. Through learning, the required features can be extracted from the complex data of the training image. Its nonlinear fitting ability is good, and its learning ability is also strong.

Because the resolution of icon symbols is very close and there is almost no noise and other interference information, there is no need to carry out pretreatment operations such as noise reduction for icon symbols.

Icon annotation is mainly used to pair similar icons in multiple training icon symbol libraries. The annotation process determines the judgment scale of model similarity. The icon symbols of forward annotation will be given a weight to the feature points extracted during model training. The influence of feature points on similarity becomes larger. In other words, if the requirements for similarity are more stringent, only the icon symbols with high similarity will be marked when labeling positive data. If the requirements for similarity are not very stringent, the standard can be reduced when labeling and the icon symbols with high similarity can also be marked.

## 4. Application of Cross Media Technology Based on Deep Learning in Landscape Performance Evaluation

### 4.1. Feature Extraction of Cross Media Landscape Based on CNN

Cross media retrieval involves two different modes of data, image and text [23]. Data preprocessing has a very important impact on the final cross media retrieval accuracy. The preprocessing of image data is relatively simple. It only needs to use RGB three-layer channels for representation, while ensuring the uniform size of the image. The representation form of text data cannot be directly used by the computer [28]. Only when the text data are converted into digital vector form can they be processed by the computer.

**Figure 6:**
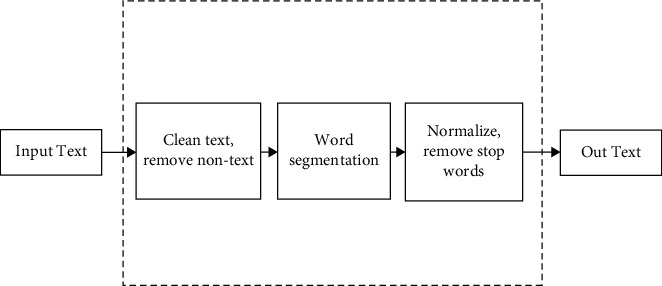
Text preprocessing process.

Text preprocessing is the premise of effective vectorization of text. After preprocessing, a lot of interference and useless information are removed, which provides a good foundation for subsequent text representation. The text preprocessing process mainly includes three steps: text cleaning, word segmentation, and standardization, as shown in Figure 6.

### 4.2. Deep Learning Processing of GIS Icon Information

CNN treats icon symbols as a 3D image matrix when processing icon symbol information. A convolution layer is a neuron connected to a small part of the neurons in the previous layer and then slides on a picture or feature block with the same connection weight as this neuron to generate another 3D neuron. A maximum pool layer is used to reduce the feature map in space. Many such layers can be stacked together in order and trained with gradient descent. This has a good effect on symbol processing.

**Figure 7:**
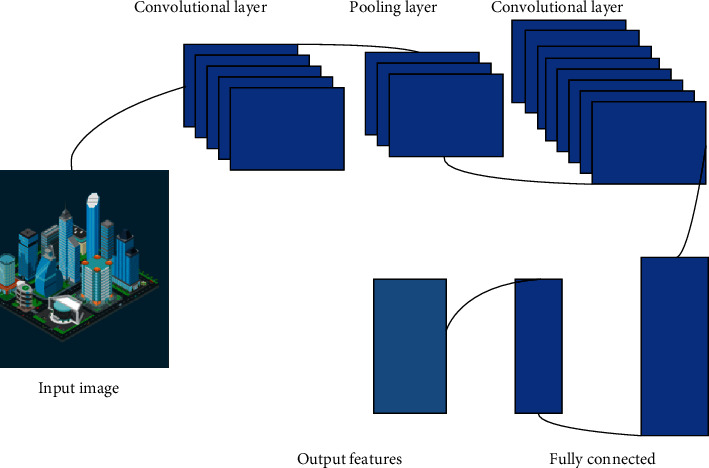
CNN handling GIS icons.

The process of image processing by CNN is the process of image disassembly, as shown in Figure 7. The leftmost image is the input image, which is gradually decomposed into more smaller images in CNN. In this process, the total number of images is increasing, and the images are decomposed smaller and smaller. The purpose of this is to decompose the complex image into a very small simple image. By searching for similarities in these simple images, the original image is derived from the very small simple image to achieve the purpose of recognition.

### 4.3. The Contribution of Deep Learning and Cross Media Retrieval to Landscape Performance Evaluation

The methods of landscape design, planning, architecture, disaster, and heritage protection are obtained by comparing the environmental and economic benefit models presented by the landscape. Based on the landscape characteristics, disaster prevention safety, cultural heritage, and humanistic sociality, this paper explores the landscape performance indicators.

According to the landscape performance evaluation system, the landscape performance evaluation index system is shown in Table 1 through consulting many studies.

## 5. Discussion

Cross media integration collects the landscape text, images, audio, and other information from multiple media. The in-depth learning represented by CNN provides richer and diversified landscape information for landscape performance evaluation. Combined with GIS technology, it effectively promotes the optimization and protection of the scenic spot landscape, attracts more tourists, enhances the popularity of the landscape, and creates social value and economic benefits.

For how to optimize the landscape performance evaluation system and create a first-class landscape, we can start from the following perspective.

Enhance the cultural benefits of landscape. The exertion of cultural artistic conception benefits from the bearing of specific materials and needs the contrast of history and culture and limited space conditions. Only when the specific material elements and other elements are conveyed as the media, can we achieve the abstract perception of the cultural artistic conception. With the transformation of space, cultural benefits will also produce dynamic, rich, and spiritual changes.

Most tourists do not have a special understanding of the cultural connotation of the landscape, and they do not have a specific cultural impression and understanding when visiting the landscape. Therefore, it is necessary to carry out science popularization and explanation in advance. The distinctive cultural image can enable tourists to form an artistic conception before visiting. The landscape can provide popular science, development history explanation, and context services, so that tourists can have a basic understanding of the ancient town culture before playing, enhance tourists' cultural identity, and stimulate tourists' exploration desire and curiosity to a certain extent.

Activate the social benefits of landscape and improve the tourism experience. The development of tourism and leisure mode is an effective way to attract tourists to “stay” in the scenic spot, and it is also an important means to prolong the life cycle of the scenic spot. Excavate the ornamental and experiential landscape culture and create projects with multiple experiences and novelty. At the same time, segment the target market population and expand the tourism market. Considering the resource characteristics and regional conditions of the scenic spot, we can further expand the tourism market by refining the tourist market.

Improve the experience quality of the landscape. We can consider using greening and landscape facilities to activate the streets and lanes of the scenic spot and create diverse landscapes. In many scenic spots, the spatial interface design is single without hierarchical changes, and the sense of spatial rhythm is weak, which cannot attract the attention of tourists. Based on such a big environment as cultural tourism and science and technology tourism, tourists' requirements for the landscape are no longer simply a cursory glance. Therefore, it is necessary to increase the science and technology of streets and lanes to attract tourists. For example, combined with local cultural characteristics, GIS and virtual technology are used to design and build a landscape rest platform for tourists to rest, relax, take photos, and punch cards, providing a buffer space for tourists to stop. Optimize the plane layout design to enhance the interest and ecology of streets and alleys. Revitalize the atmosphere of streets and lanes and improve the quality of tourist experience in the town.

## 6. Conclusion

Internet users are the makers of multimedia data. Data such as landscape images, texts, and videos displayed in multiple platforms based on GIS technology gush out. The scenic area will publicize the scenic area through text, pictures, videos, route display, geographical location annotation, scenic area evaluation system, experience evaluation, and other ways and conduct landscape performance evaluation. The cross media retrieval technology based on deep learning effectively solves the problems caused by the trend of information diversification. Cross media fusion technology includes computer vision, artificial intelligence, machine learning, pattern recognition, and other fields. It provides a more intelligent processing means and data collection platform for the construction of landscape performance evaluation system and data screening. It enriches the data sources of landscape samples and constructs the performance landscape evaluation system in a more clear and accurate way.

In future, we will devote ourselves to exploring and optimizing more in-depth learning models to improve the efficiency of neural network in retrieving and processing landscape data and build a unique landscape performance evaluation system.

## Figures and Tables

**Table 1:** Landscape performance evaluation system.

Primary indicator	Secondary indicator	Explanation of indicator
Landscape characteristics	Location	The reasonableness of the landscape environment
	Spatial pattern	Design concept displayed in landscape space and architectural space
	Stone and water	Rock and water design rationality and degree of protection
	Vegetation	The rationality of plant configuration and the degree of protection
	Architectural style	Architectural details and coordination
	Environmental coordination	Degree of coordination with the surrounding environment

Disaster prevention safety	Fire	Fire prevention ability
	Flood	Flood prevention ability
	Man-made accident	Preventive measures against man-made accidents
	Noise	Sound environment quality
	Other disasters	Preventive measures against man-made accidents

Cultural heritage	Cultural	Local customs and artistic hierarchy
	Historical features	History background

Humanity and sociality	Tourist satisfaction	Tourist recognition
	Entertainment and social value	Entertainment and social role
	Job opportunity	Employment opportunities for surrounding residents

## Data Availability

The dataset used in this paper is available from the corresponding author upon request.
